# Making better use of our brain MRI research data

**DOI:** 10.1007/s00330-012-2408-3

**Published:** 2012-03-17

**Authors:** Frederik Barkhof

**Affiliations:** Department of Radiology, VU University Medical Center, Amsterdam, The Netherlands

Increasingly, MRI is used outside the diagnostic setting to study brain physiology, or the biological mechanisms of brain maturation and ageing. In an era with an increasingly ageing population, the study of age-related brain changes is important to understand risk factors and early signs of neurodegenerative disease, such as Alzheimer’s disease. To detect subtle changes in brain structure, large image banks are typically needed, for example to examine the impact of rare or weak genetic traits. Those studies often require substantial efforts to collect and then are used to address a limited set of questions framed by the original investigators, even though they would allow additional questions to be considered by others if disclosed publically and well documented. One could argue that publicly funded agencies of such studies would get better value for the money if the data were used by more than one research group, a trend that is now being translated into new policies regarding ownership of research material in various countries. The ADNI project (www.adni-info.org) was a big step forward, where the ultimate goal was to create a publically accessible data set, which to date has already been used in hundreds of published studies. One advantage of such a prospective initiative is that the homogeneity of images and metadata is better than for retrospectively disclosed repositories, with often only partly overlapping data dictionaries.

The review by Dickie et al. [1] in the current issue systematically reviews open access of publically accessible databanks of the ageing brain. Among a large number of potential databases, only a few were found to have public access; among those, nine fulfilled the inclusion criteria, and far less than 1,000 complete MR imaging data sets were available in subjects over the age of 60. Furthermore, access is often not easily obtained (even for ADNI user have to submit a proposal and obtain approval); metadata (e.g., clinical) are often presented incompletely (and vary between studies), intermediate results (e.g., segmentations or statistical maps) are typically lacking and basic MRI descriptors (e.g., the amount of white matter lesions) not available. Therefore, despite the excellent original intentions, the yield of public databases so far has been modest.

What would be needed to enhance to unique possibilities in this field? First of all, the need for a public call to disclose more data publically should be articulated. The paper by Dickie et al. [1] helps to revive this public appeal. Secondly, there will be a need for a consortium that proactively approaches potential data donors. For example, the MRI data from existing studies such as the Cardiovascular Health Study (www.chs-nhlbi.org) or the Rotterdam Scan Study are not available, although they were funded by government money. Thirdly, for major funding requests, public funding agencies should strongly prefer applications that have both the plan and commitment to make the data publicly accessible within 2 or 3 years of acquisition (making the sponsors rather than the investigators the owners of the data). Fourthly, an independent forum may be needed to share the data publically in a regulated environment, ensuring subject confidentiality and data integrity. For example, the FP7 project entitled neuGRID (www.neugrid.eu) provides such a portal, and at the same time allows users to take advantage of the virtual laboratory and computing facilities typically needed to handle such large image data sets [2]; similar endeavours exist in North America (www.outgrid.eu). Fifthly, to ensure academic independence, users of the data should only have to report published results to the original investigators. Sixthly, there should be standards of reporting data and storing of intermediate results that can be used by subsequent users, and avoid replication of for example segmentation and registration efforts. Finally, metadata such as clinical, laboratory, genetic and other information should be unified. Standard terminology in various domains is being developed by the NINDS (http://www.commondataelements.ninds.nih.gov).

Future expansion beyond the study of normal ageing could include disease states, such as Alzheimer's, or into other imaging techniques, such as nuclear medicine. Beyond the collection of anatomic brain MRI scans, there is increasing interest in more advanced MRI data, such as resting-state functional MRI and diffusion tensor imaging (DTI) to unravel the “human connectome” (http://www.humanconnectomeproject.org and humanconnectome.org). An example of PET/SPECT data sharing in Alzheimer’s disease already exists (https://www.eu-decide.eu). Data sharing could also be expanded to other disease areas where international collaborations already exist, such as for multiple sclerosis (www.magnims.eu) and Huntington's disease (http://hdresearch.ucl.ac.uk/our-results/track-hd), where currently such data are usually not publicly accessible.

The scale of economy that is needed in brain imaging research calls not only for larger studies in ageing and dementia, but also for better use of data by third parties by making such data publicly accessible. Ideally, this is considered at the study outset, allowing interoperability of images and metadata with existing repositories.
